# Monitoring of omalizumab therapy in children and adolescents 

**DOI:** 10.5414/ALX01337E

**Published:** 2018-09-01

**Authors:** J.O. Steiß, A. Schmidt, H. Lindemann, S. Rudloff, A. Staatz, P. Strohner, G. Becher, L. Nährlich, K.P. Zimmer

**Affiliations:** 1 Zentrum für Kinderheilkunde und Jugendmedizin, Justus-Liebig-Universität Gießen,; 2 BioTeZ Berlin-Buch GmbH,; 3 BecherConsult GmbH Berlin

**Keywords:** anti-IgE, asthma, immunoglobulin E, omalizumab therapy, therapeutic monitoring

## Abstract

Background: Omalizumab is a successfully implemented supplementary therapy for improving asthma control in children aged 6 years and older with severe persistent allergic asthma. The dosage of omalizumab depends on body weight and IgE level, yet no parameter has been established to guide dosage changes during therapy. Clinical studies in patients with allergic asthma or allergic rhinitis revealed a clinically relevant improvement by using omalizumab leading to concentrations of free serum IgE reported to be lower than 50 ng/ml. Therefore, only the question concerning the concentrations of free IgE used in a therapy with omalizumab is regarded of clinical importance, while total IgE (free and omalizumab-bound IgE) increases during treatment. Patients and methods: Ten patients, 8 to 17 years of age, received therapy with omalizumab due to severe allergic asthma. In addition, the patients had pronounced rhinoconjunctivitis, food allergy, insect sting allergy, and/or neurodermitis. The total IgE in the serum was measured in the patients 3 – 6 months before each omalizumab injection as a potential progress parameter (Sandwich-Immunoassay ADVIA Centaur). Results: Six months after beginning of the therapy with omalizumab, a significant decrease of the total IgE concentration was found, in comparison to the baseline values (p < 0.003). In all patients the tolerability of omalizumab was very good: there was a reduction in the frequency of the asthma exacerbations and rescue medications. All patients reported a clearly improved quality of life. Conclusions: A general increase in IgE was not observed in any of the children we treated with omalizumab. Apart from the development of routine assays to determine free serum IgE levels, the significance of the total serum IgE as a suitable control of an omalizumab therapy should be further investigated in controlled studies with regard to sensitivity and specificity. In order to only administer the lowest necessary dose of omalizumab especially in children and adolescents, the establishment of laboratory parameters (free IgE and/or total IgE) to adequately monitor the therapy is urgently needed. Patients undergoing an omalizumab therapy require medical supervision at close intervals.

**German version published in Allergologie, Vol. 33, No. 10/2010, p. 421-428**

## Introduction 

The prevalence of severe bronchial asthma in children and adolescents has been growing over the past decades. In Germany approximately 400,000 children are affected, which corresponds to approximately 10% of asthma patients. In at least 70% of cases the symptoms develop before the child is 5 years old. An allergic pathogenesis is present in about 70% of asthmatic children, many of whom are still inadequately treated [2, 6, 21]. 

Since 2005 the humanized antibody omalizumab is available as a supplementary therapy for improved asthma control in patients with severe persisting allergic asthma. Omalizumab is a monoclonal anti-IgE antibody that binds to circulating IgE molecules and suppresses the expression of the high-affinity IgE receptor (FcεRI) on mast cells, basophil granulocytes and other immunologic effector cells [8, 12]. Dosage and frequency of omalizumab application depend on total serum IgE at baseline and body weight (Table 1a, Table 1b). The total IgE concentration seems to be increased after administration of omalizumab, as this value includes both free IgE and omalizumab-bound IgE [9]. So far, only the reduction of asthma symptoms and the improvement of asthma control are used to evaluate the course in patients treated with omalizumab. Although there is evidence that both omalizumab and free serum IgE concentrations correlate with changes of clinical parameters induced by omalizumab therapy, no routine method is available so far that would determine free serum IgE in order to monitor anti-IgE therapy [14, 19]. 

## Patients and methods 

We report on 10 patients aged 8 – 17 years with severe allergic bronchial asthma, partially with concomitant pronounced rhinoconjunctivitis (n = 8), peanut allergy (n = 2), insect venom allergy (n = 1) and/or atopic dermatitis (n = 4), in whom we decided to start therapy with omalizumab (Table 2). Within the last 3 months before omalizumab therapy the patients had been on continuous therapy with high-dose inhaled steroids ( 400 mg budesonide equivalent or 200 mg fluticasons, respectively), some of them in combination with a long-acting beta agonist (LABA) and/or leukotriene antagonist (LTRA), or on therapy with systemic steroids. All patients were treated due to increased total IgE concentrations, partially outside the range of the then valid dose recommendations (n = 7), and/or due to their age of < 12 years (n = 4) after written informed consent of their parents. Subsequently, total serum IgE was measured in intervals of 3 – 6 months before omalizumab injection (ADVIA Centaur Sandwich immunoassay). Evaluation of therapy success was carried out using the following parameters: total therapy evaluation by the physician in consideration of information given by the patients, PEF (peak expiratory flow) and frequency of exacerbations. 

## Results 

**Figure 1.**
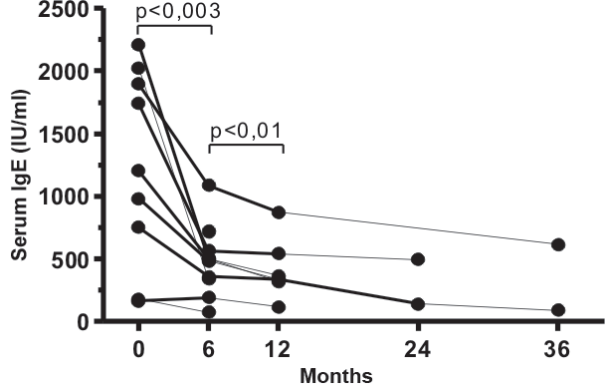
Total IgE in 10 children with severe bronchial asthma in the course of omalizumab therapy.

**Figure 2.**
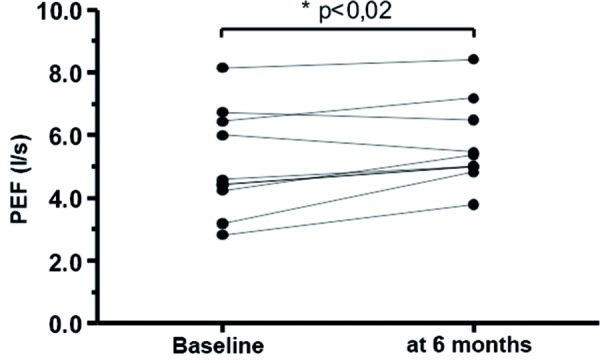
PEF (l/s) before and 6 months after start of omalizumab therapy in 10 children with severe bronchial asthma.

Six months after onset of omalizumab therapy a marked reduction in total IgE concentration was detectable in 9 of the 10 patients. In 1 patient this reduction was only present from the 9^th^ month on. The reduction of total IgE was accompanied by a clinical improvement reported by the patients. The mean concentration before start of omalizumab therapy was 1,372.8 ± 845.1 IU/ml, after 6 months of therapy it was 481.6 ± 281.6 IU/ml (p < 0.003) (Figure 1). This trend continued in the further course of therapy (p < 0.01) (Figure 1). In none of the patients an increase in total IgE was detected. 2 – 3 years after start of therapy the total serum IgE values were partially within the normal range (Figure 1). In all patients the tolerability of omalizumab was very good, and there was a reduction in the frequency of asthma exacerbations (Table 2), need for rescue medication and dose of inhaled glucocorticoids. Pulmonary function tests should an improved PEF after 6 months of omalizumab therapy (5.1 l/s ± 1.7 before therapy; 5.7 l/s ± 1.3 after 6 months of therapy; p < 0.02) (Figure 2). Concerning comorbidities some differences between the patients were observed. Patients with peanut allergy tolerated chocolate with low amounts of nuts under anti-IgE therapy. With regard to atopic dermatitis the results under omalizumab therapy were less impressive: in 2 of the 4 patients the skin symptoms improved, while in the other 2 patients no improvement of atopic dermatitis was observed. All patients reported a clearly improved quality of life and capacity to participate in activities. They could, for example, participate in more sporting activities, were less frequently absent from school and did not have to be admitted to hospital. Although no systemic steroids were administered during omalizumab therapy, asthma symptoms did not worsen. 

## Discussion 

Omalizumab is used as supplementary therapy for the improvement of asthma control in patients with severe persisting allergic bronchial asthma who show positive skin tests or in vitro reactivity against a perennial airborne allergen and suffer from frequent symptoms during the day or from nocturnal awakening and experience exacerbations despite therapy with high-dose inhaled corticosteroids and a long-acting β_2_-agonist. Since 2009 this therapy has also been approved for children from the age of 6 years on and recently also for total serum IgE concentrations of up to 1,500 IU in a maximum dose of 1,200 per month. Numerous case reports also confirm the efficacy of omalizumab in patients with higher IgE levels [15, 16, 25]. Different studies have proven that omalizumab is safe and well-tolerated in children [4]. In the current randomized, double-blind, placebo-controlled, multicenter pivotal study in children aged 6 to < 12 years a reduction of exacerbations under omalizumab therapy was detected in 31% of patients (p < 0.007). Adverse events that were presumably related to the drug were reported in 6.9% of patients in the omalizumab group and in 8.8% of patients in the placebo group. The most frequently reported adverse events were headache, erythema, injection site reactions, urticaria, nausea and tremor [17]. Thus, the safety profile of omalizumab in children corresponds to the experience made in other age groups [13, 20]. 

Depending on its dose, anti-IgE leads to a fast reduction of free IgE in the serum and reaches its highest serum concentration after a mean of 7 – 8 days [7, 11]. Clinical trials could show that a relevant improvement is induced by omalizumab at a free serum IgE level of 50 ng/ml [1, 5]. Despite this effect, increases in total serum IgE have been described, resulting from the combination of free IgE and IgE-containing immune complexes. In an often-cited study by Hamilton et al. [9] an increase in total IgE has been shown by ImmunoCAP 250 (Pharmacia, Kalamazoo, MI, USA) in a total of 12 patients. The reason is suspected to be the elimination of complexed IgE with a half-life of approximately 2 weeks over the reticulohistiocytic system of the liver. Furthermore, most of the commercially available assays seem to bind IgE to another epitope than omalizumab. This means that free as well as complexed, i.e. omalizumab-bound, IgE is measured [3]. In addition to objective parameters like lung function, quality of life and symptom diaries, the physician’s global evaluation was also included in the therapy monitoring. In this context it was revealed that patients who were classified as responders by the physicians showed a more pronounced improvement than the non-reponders [4]. This suggests that the subjective evaluation made by the physician is currently the most reliable instrument for the monitoring of omalizumab therapy. 

Omalizumab is dosed according to body weight and total IgE. Although, despite this seemingly very rough dose specification, the efficacy of anti-IgE therapy has been well documented in numeruous studies in large patient collectives, single cases show a divergency concerning the effect of omalizumab [26]. It is suspected that the effect depends on the availability of free IgE antibodies in the serum. The reduction of free serum IgE levels is associated with a reduced expression of FcεRI receptors on mast cells and basophil granulocytes. Thus, several researchers are trying to find a method to quantify free IgE during omalizumab therapy in order to allow for the fine tuning of anti-IgE therapy in each individual patient. 

In all our patients clinical efficacy was shown by a reduction of the frequency and severeness of exacerbations, less presentations at the emergency unit and hospital admissions as well as by an improved asthma control and quality of life. In the context of pulmonary function parameters in children it has to be taken into account that criteria used for adults frequently cannot be transferred to children, because in some pediatric patients with severe asthma the lung function values at rest are still in the “normal” range. Thus, a clinically unquestionable obstruction can be present and needs to be treated even if lung function values seem to be “normal” (FEV_1_ > 80% and MEF_50_ > 65%). Also PEF seems to be of only limited value as a parameter for the control of omalizumab therapy. 

An increase in total IgE under omalizumab therapy, as it was described in the literature, was not detectable in any of our pediatric patients [22]. On the contrary, in some patients a reduction to normal ranges was achieved. Also other case reports show a reduction in total IgE under omalizumab therapy [27]. The ADVIA-Centaur total IgE test that we used for our assessments is a two-pronged direct chemiluminescence sandwich immunoassay using constant amounts of two IgE antibodies. The first antibody (lite reagent) is a goat anti-human IgE-antibody marked with acridinium ester. The second antibody (solid phase) is a mouse anti-human IgE antibody which is covalently bound to paramagnetic particles. There is a directly proportional relationship between the amount of total IgE in the patient sample and the relative units of light measured by the system. We suspect that in the ADVIA-Centaur assay that we used the human IgE binds to the same epitope as omalizumab. 

The clinical observation of a reduction in total IgE is supported by Lowe et al. [18]. Using a direct binding model that included dissociation constants and kinetic parameters for omalizumab, IgE and omalizumab-IgE complexes, these authors could calculate from the data of 1,682 patients with allergic asthma or rhinitis who were included in a total of four clinical omalizumab studies that omalizumab could reduce IgE production to the normal rate of non-atopics in the long term. Thus, omalizumab is able to normalize IgE production in patients with moderate-to-severe atopic asthma [18]. In 2007 Hanf et al. [10] reported a reduction of IgE secretion and circulating B-lymphocytes under omalizumab therapy. 

The isolated determination of free IgE in a “recovery ELISA” is based on the conventional principle of a sandwich immunoassay. In a “recovery ELISA”, which is still not available for routine use, the concentration of an antigen is determined by two antibodies (the so-called catcher and signal antibody, respectivley) that are directed against epitopes of this antigen. An antibody pair consisting of catcher and signal antibodies can only be produced for antigens that are large enough and thus possess different epitopes. The signal antibody is usually used in a “labeled” form, i.e. the signal antibody possesses a specific molecule (label) that allows to detect a reaction. The difference to the conventional sandwich assay is that in an R-ELISA the therapeutic antibody, or a labeled antibody that possesses the same binding epitope as the therapeutic antibody, is used as a signal antibody. If the therapeutic antibody is also present in the patient sample, but in an unlabeled form, this results in a systemic reduction of the recovery of the antigen in question correlating with the concentration of the therapeutic antibody. This correlation can be used to determine the unknown concentration of the therapeutic antibody and to evaluate the free and total antigen concentration [23, 24]. 

It is essential to develop a routine therapy monitoring procedure to make sure that only the necessary dose of omalizumab is applied, particularly for long-term therapies in chilrend and adolescents. Now that we have been using omalizumab very successfully for almost 5 years we have to find out the appropriate dose for children. An increase in total IgE under omalizumab therapy does not seem to be a general observation. A long-term omalizumab therapy that is exclusively based on baseline body weight and total IgE is too inexact and cannot cope with the complex mechanisms of omalizumab. Apart from the development of routine assays to determine free serum IgE levels, the significance of the total serum IgE as a suitable control of an omalizumab therapy should be further investigated in controlled studies with regard to sensitivity and specificity. Patients undergoing an omalizumab therapy require medical supervision at close intervals. 

## Conflict of interest statement 

Jens-Oliver Steiß received financial support for attending congresses and honoraria for conference speeches from Novartis. 

**Table 1a.**
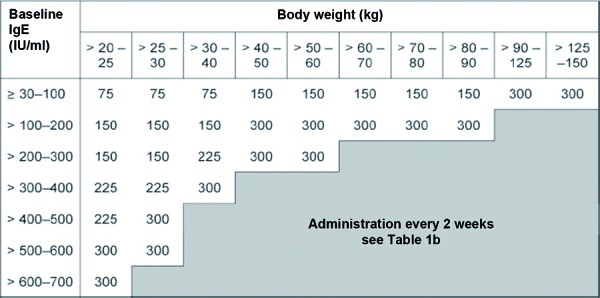
Omalizumab dosage every 4 weeks (mg/dose) based on total serum IgE at baseline and body weight.

**Table 1b.**
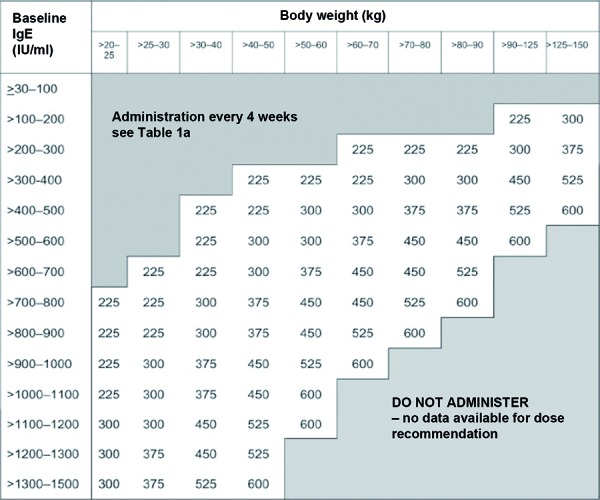
Omalizumab dosage every 2 weeks (mg/dose).

**Table 2.** Patients on omalizumab therapy.

No.	Age/ Gender	Diagnosis	Dose (mg) every 2 – 4 weeks	IgE (IU/ml) Base- line	IgE at 6 months	IgE at 12 months	IgE at 24 months	IgE at 36 months	Exacer- bations/ year before start of therapy	Exacer- bations/ year after 12 months
1	14 m	asthma IV rhinoconjunctivitis	750/4 W	982.0	481.6	339.8			5	0
2	13 m	asthma IV rhinoconjunctivitis, atopic dermatitis	600/4 W	2,575.0	719.3				0.1 mg/ kg BW steroid as continous treatment	0
3	17 f	asthma IV	600/4 W	2,023.0	338.6				5	3
4	8 f	asthma III – IV	150/4 W	179.2	75.3				3 – 4	0
5	11 f	asthma III – IV rhinoconjunctivitis, atopic dermatitis, food allergy	300/2 W	1,896.0	1,086.0	873.9		615.8	3 – 4	0
6	11 f	asthma IV rhinoconjunctivitis, insect venom allergy, atopic dermatitis, food allergy	300/2 W	754.1	358.5	337.0	138.7	89.6	5 – 6	0
7	15 m	asthma III – IV Rhinoconjunctivitis	450/2 W	2,210.0	500.8	321.4			2 – 3	1
8	12 f	asthma IV rhinoconjunctivitis, atopic dermatitis	300/2 W	1,741.0	566.0	541.8	495.0		3 – 4	1
9	10 m	asthma III – IV rhinoconjunctivitis, polyvalent sensitization	300/2 W	1,204.0	496.4	368.6			4	1
10	13 m	asthma III – IV rhinoconjunctivitis, polyvalent sensitization	300/2 W	164.6	193.1	115.8			4	0
